# SEL1L3 as a link molecular between renal cell carcinoma and atherosclerosis based on bioinformatics analysis and experimental verification

**DOI:** 10.18632/aging.205227

**Published:** 2023-11-21

**Authors:** Haoyuan Wang, Xiaopeng Ma, Sijie Li, Xiaochen Ni

**Affiliations:** 1 Department of Urology Surgery, The Fourth Hospital of Hebei Medical University, Shijiazhuang 050011, Hebei Province, China; 2 Hebei Medical University, Shijiazhuang 050011, Hebei Province, China

**Keywords:** renal cancer, SEL1L3, atherosclerosis, potential biomarker, bioinformatics

## Abstract

Background: Renal cancer, the most common type of kidney cancer, develops in the renal tubular epithelium. Atherosclerosis of the aorta is the primary cause of atherosclerosis. However, the underlying mechanisms remain unclear.

Methods: The renal clear cell carcinoma RNA sequence profile was obtained from The Cancer Genome Atlas (TCGA) database, and the atherosclerosis datasets GSE28829 and GSE43292 based on GPL570 and GPL6244 was obtained from the Gene Expression Omnibus (GEO) database. The difference and hub genes were identified by the Limma protein-protein interaction (PPI) network in R software. Functional enrichment, survival, and immunoinfiltration analyses were performed. The role of SEL1L3 in the ErbB/PI3K/mTOR signaling pathway, apoptosis, invasion, cell cycle, and inflammation was analyzed using western blotting.

Results: 764 DEGs were identified from TCGA Kidney Renal Clear Cell Carcinoma (KIRC) dataset. A total of 344 and 117 DEGs were screened from the GSE14762 and GSE53757 datasets, respectively. Functional enrichment analysis results primarily indicated enrichment in the transporter complex, DNA-binding transcription activator activity, morphogenesis of the embryonic epithelium, stem cell proliferation, adrenal overactivity and so on. Fifteen common DEGs overlapped among the three datasets. The PPI network revealed that SEL1L3 was the core gene. Survival analysis showed that lower SEL1L3 expression levels led to a worse prognosis. Immune cell infiltration analysis showed that SEL1L3 expression was significantly correlated with antibody-drug conjugates (aDC), B cells, eosinophils, interstitial dendritic cells (iDC), macrophages, and more.

Conclusions: SEL1L3 plays an important role in renal clear cell carcinoma and atherosclerosis and may be a potential link between them.

## INTRODUCTION

Renal cell carcinoma (RCC) is the largest pathological subtype of adult kidney cancer and is among the top ten most common cancer types worldwide [1, 2]. Because it is insensitive to traditional radiotherapy and chemotherapy, surgical treatment still is the first-line option for localized renal cancer therapy [3]. Because of the asymptomatic nature of RCC and the lack of effective early diagnostic markers, approximately 30% of patients develop distant metastases [3–5]. Metastatic RCC is mainly treated with targeted drugs, such as sorafenib and sunitinib. The high cost of these drugs and the problems created by prolonged use of these drugs, such as drug resistance, toxicity and side effects, restrict their use in clinical practice [3–5]. Therefore, gaining insight into the mechanisms underlying RCC progression and identifying novel biomarkers and molecular targets for RCC are urgently required.

Atherosclerosis is a persistent inflammatory condition that affects the arteries and is characterized by the accumulation of lipids within the walls of blood vessels, leading to the development of atherosclerotic plaques [6, 7]. It is widely believed to be the principal etiological factor that contributes to cerebrovascular disorders. Advanced carotid atheroma has the potential to give rise to unstable plaques with a propensity for rupture, thereby constituting the primary origin of local thrombosis or emboli [8, 9]. It is more important to prevent the formation of unstable plaques than to intervene in existing unstable plaques. Therefore, it is essential to understand the inherent molecular mechanisms and mine new therapeutic targets.

Progress in high-throughput sequencing technology has led to the development of sophisticated data-mining techniques for the analysis of high-throughput DNA sequencing data [10, 11]. Bioinformatics technology is an interdisciplinary field [12]. Bioinformatics plays a pivotal role in tumor treatment through the elucidation of the biological significance of vast datasets, thereby bridging the gap between data and clinical applications. Specifically, the analysis and reporting of gene detection data exemplify the essential contributions of bioinformatics in this context [13].

SEL1L family member 3 (SEL1L3) is involved in the pathogenesis of various human malignancies [14–17]. However, the relationship between RCC and atherosclerosis remains unclear. To elucidate the connection between RCC and atherosclerosis, this study aimed to identify SEL1L3 as a pivotal gene influencing both conditions and subsequently validate these findings through an experimental approach.

## RESULTS

### Differential gene expression analysis

**Figure 1:**
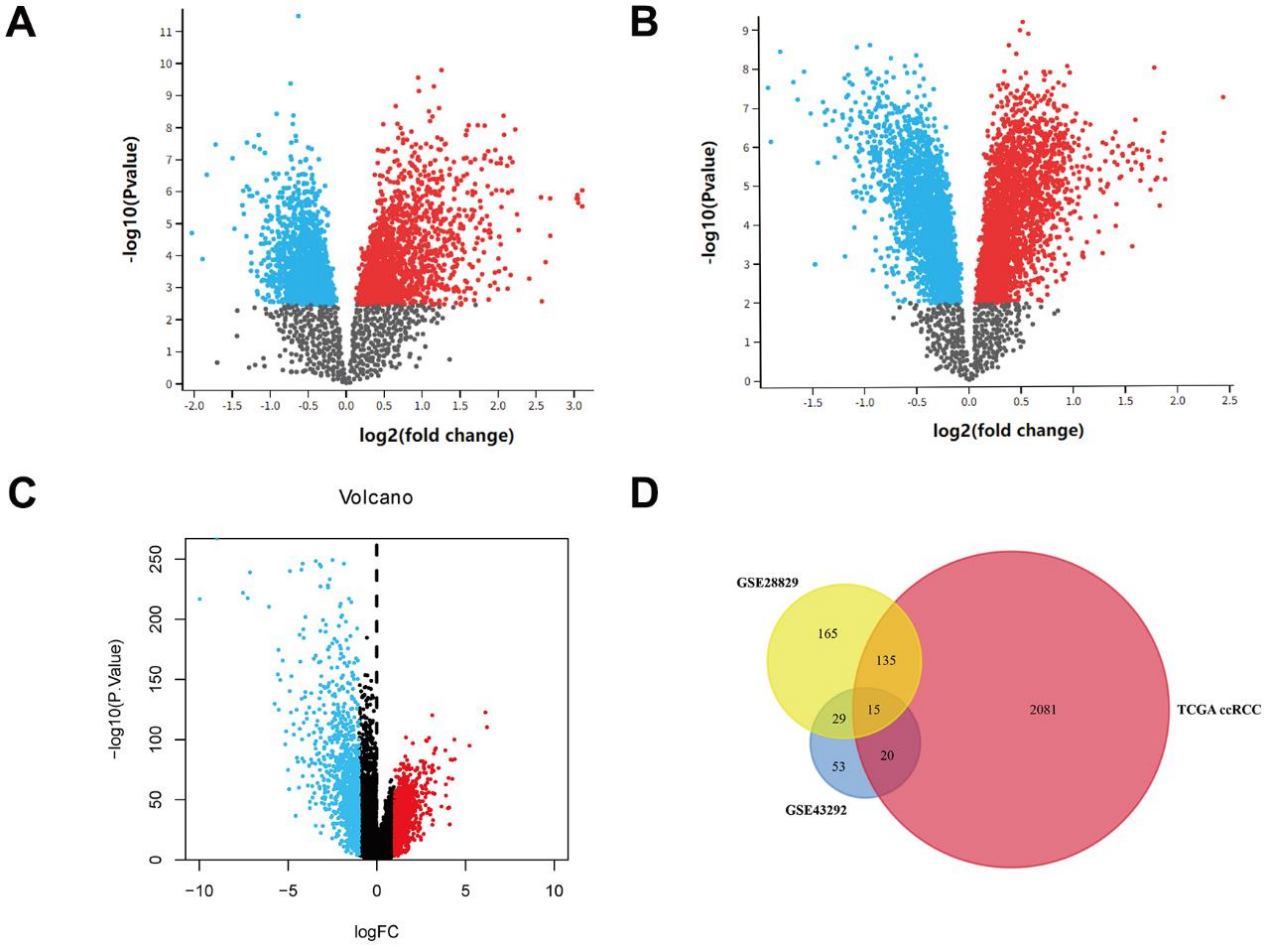
**Identification of DEGs.** (**A**) Volcano plot of GSE14762. (**B**) Volcano plot of GSE53757. (**C**) Volcano plot of TCGA KIRC dataset. (**D**) Venn plot of three datasets.

In total, 344 and 117 DEGs were found in GSE14762 and GSE53757, respectively, according to the pre-set cutoff values (Figure 1A, 1B). A total of 764 DEGs were screened from TCGA Kidney Renal Clear Cell Carcinoma (KIRC) dataset (Figure 1C). By overlapping the DEGs from the three datasets, 15 DEGs were identified (Figure 1D).

### Functional enrichment analysis

### GSEA

**Figure 2:**
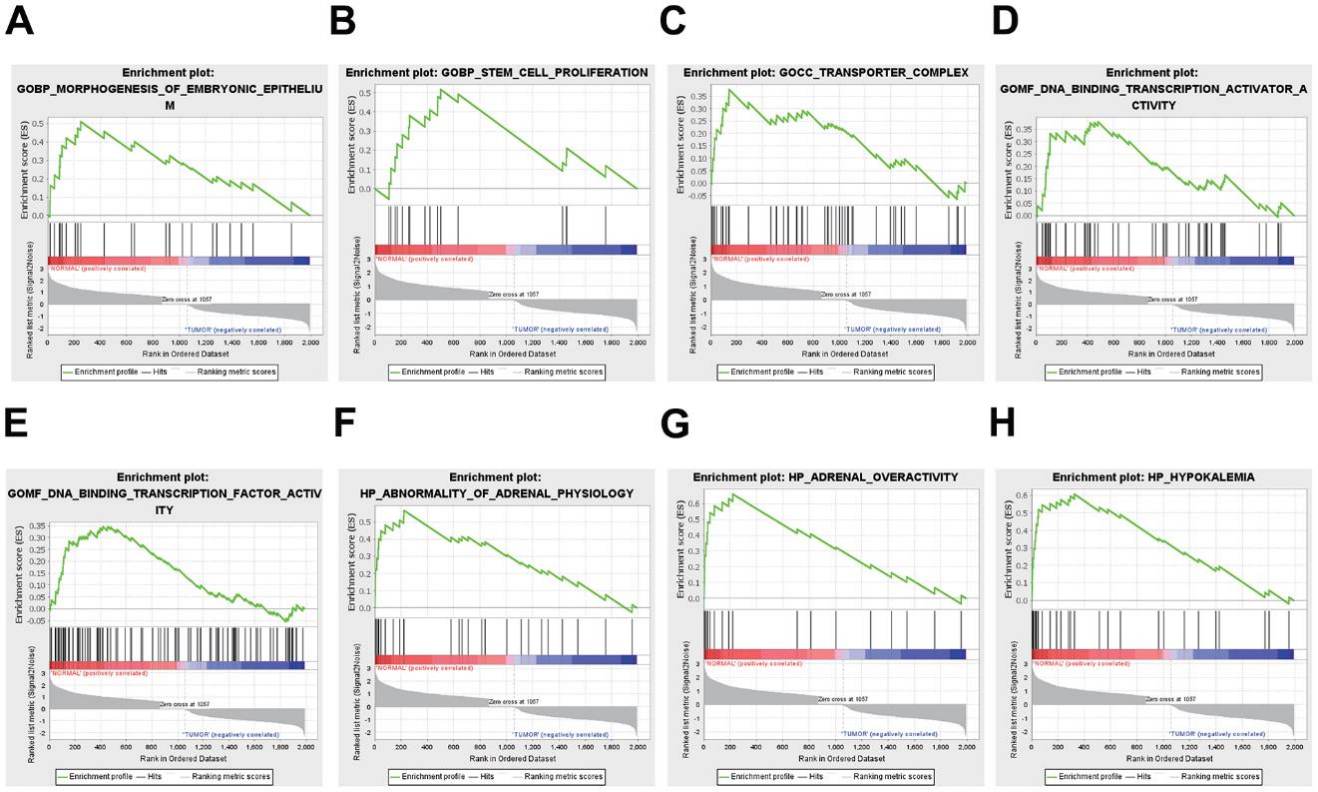
**Functional enrichment analysis results from GSEA.** (**A**, **B**) BP, (**C**) CC, (**D**, **E**) MF, (**F**–**H**) HP analysis by GSEA.

GSEA was used to conduct a comprehensive genome-wide enrichment analysis (GWEA). The prominent enriched pathways included DNA-binding transcription activator activity, transporter complex, morphogenesis of embryonic epithelium, stem cell proliferation, adrenal overactivity, DNA-binding transcription factor activity, abnormalities in adrenal physiology, and hypokalemia (Figure 2).

### Metascape enrichment analysis

**Figure 3:**
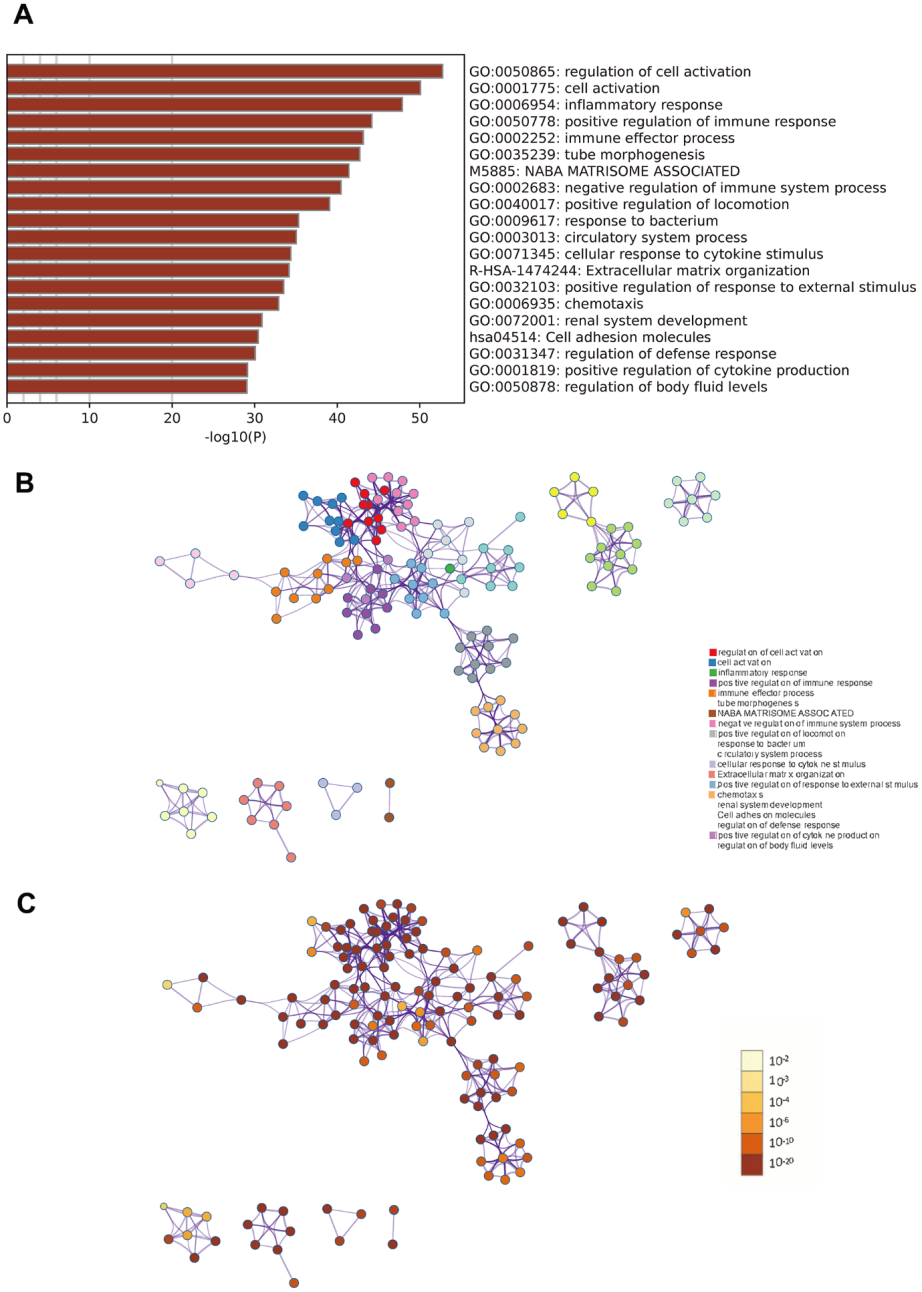
**Metascape enrichment analysis.** (**A**) Heatmap of enriched terms across input differently expressed gene lists, colored by p-values. (**B**) Network of enriched terms colored by cluster identity. (**C**) Network of enriched terms colored by p-value.

Metascape analysis revealed strong enrichment in the regulation of cell activation, inflammatory response, and positive regulation of immune response, immune effector processes, and tube morphogenesis (Figure 3).

### PPI network

**Figure 4:**
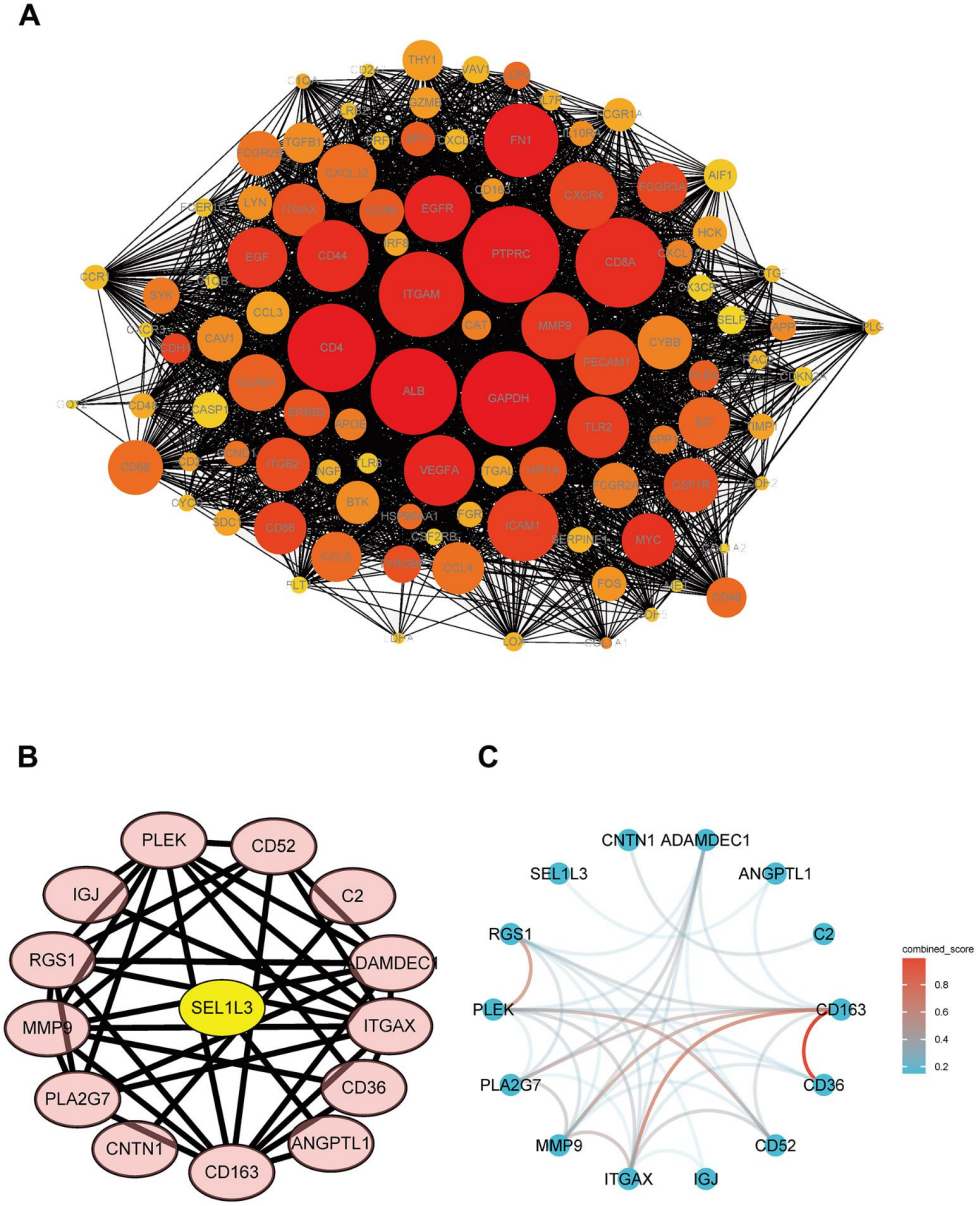
**PPI network of DEGs and hub genes.** (**A**) Total DEGs. (**B**) Protein interaction relationship by Cytoscape. (**C**) Protein interaction network colored by combine score.

Cytoscape software was used to visualize the PPI network. The total numbers of DEGs in the three datasets (Figure 4A). The connections among the 15 common DEGs (Figure 4B). Among the DEGs, SEL1L3 was identified as the core gene (Figure 4B).

### Survival analysis

**Figure 5:**
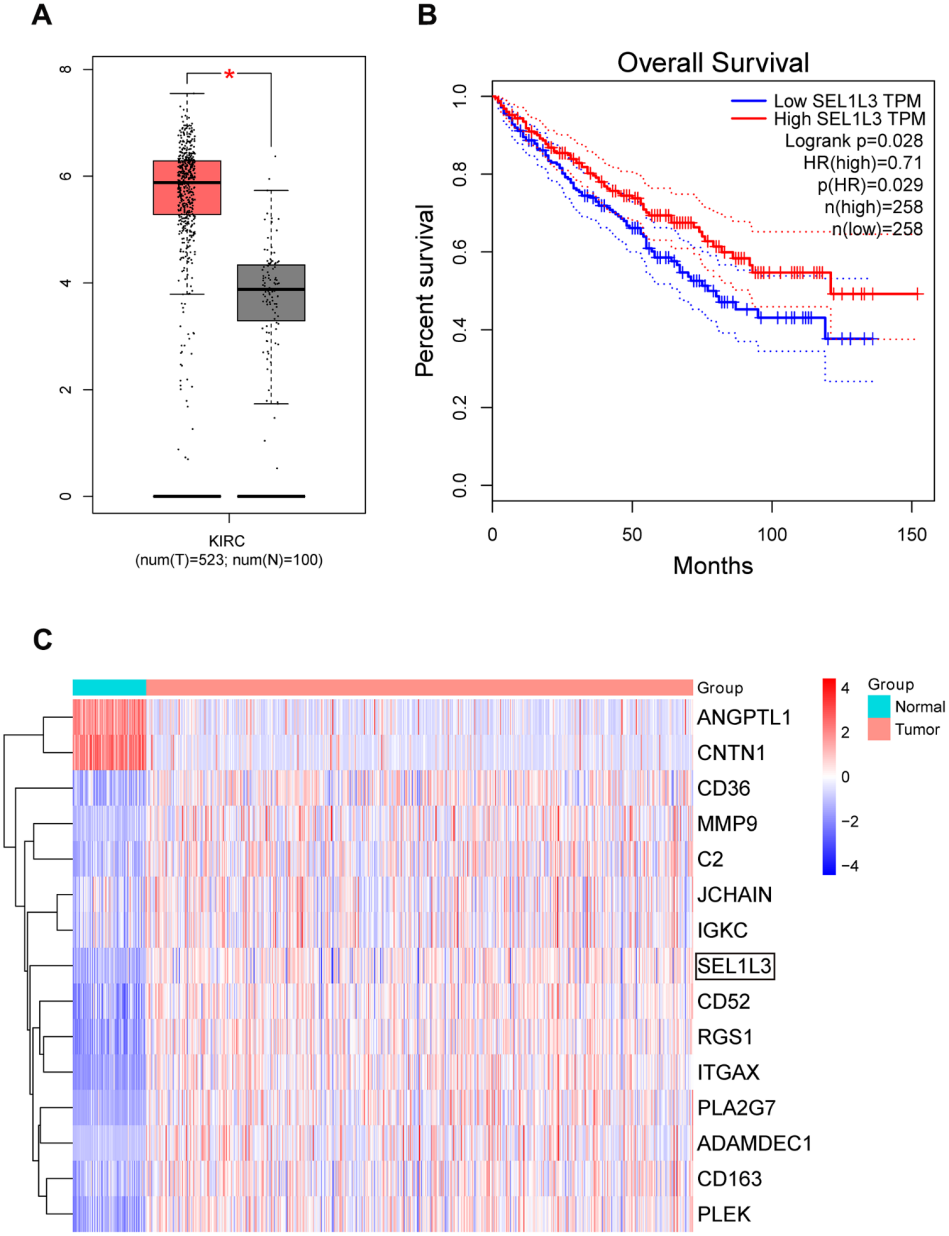
(**A**) Expression box plot of SEL1L3 in TCGA KIRC dataset. (**B**) Survival curve of SEL1L3 in KIRC dataset. (**C**) Heatmap of DEGs.

SEL1L3 expression was significantly higher in RCC samples than in normal samples (Figure 5A). Patients with lower SEL1L3 expression had poorer overall survival (log-rank p =0.028, Figure 5B).

### Gene expression heatmap

A gene expression heatmap was used to visualize (Figure 5C). It was observed that the SEL1L3 gene exhibited high expression levels in RCC samples, whereas it demonstrated low expression levels in normal tissue samples. This observation suggests a potential regulatory involvement of SEL1L3 in these cancer types.

### Immune infiltration analysis

**Figure 6:**
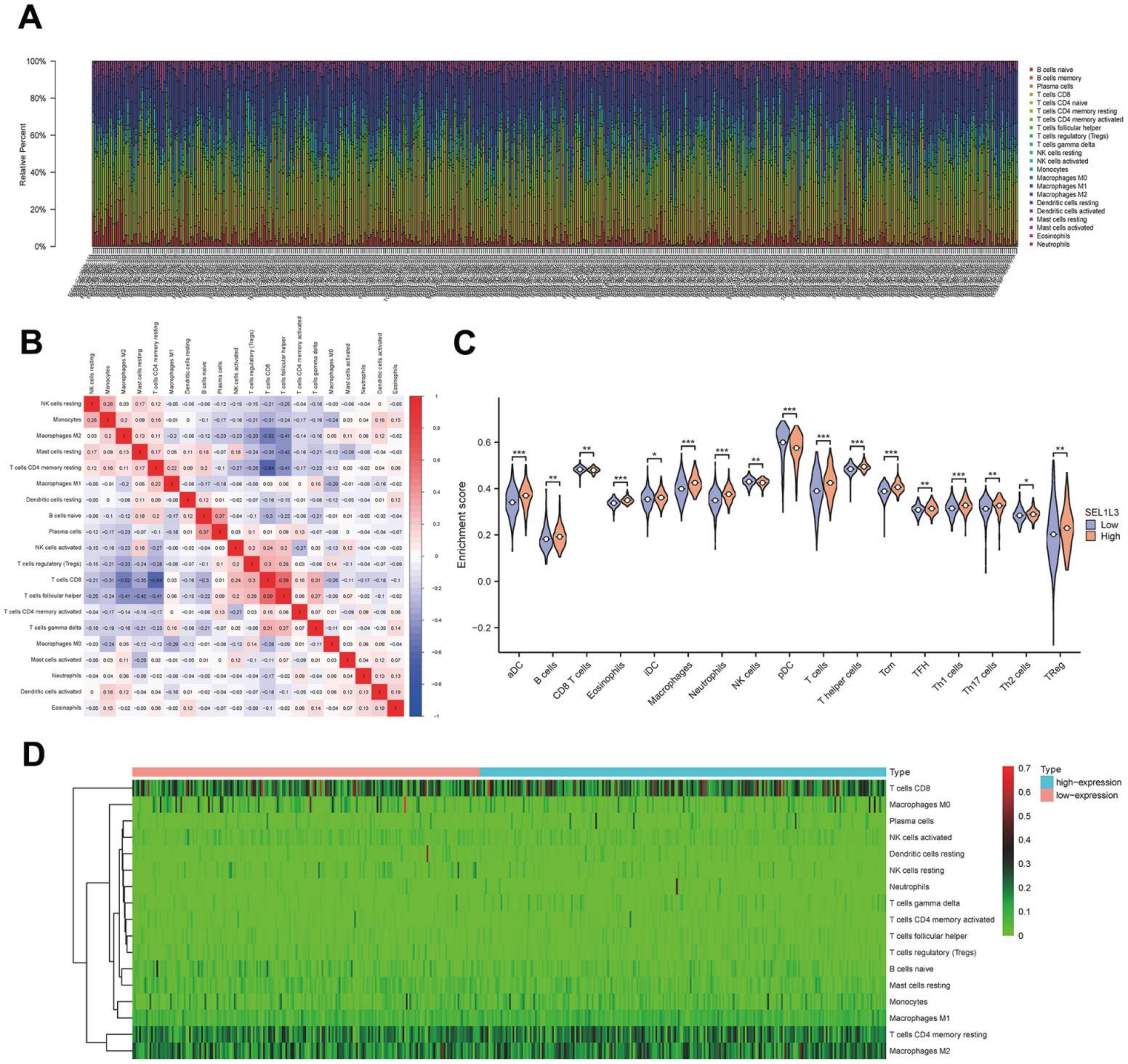
**Immune infiltration analysis.** (**A**) Fractions of immune cells in whole KIRC sample. (**B**) The correlational heatmap of 20 difference immune cells. (**C**) Violin plots of the distribution of difference immune cells. (**D**) Calorigram reflecting the distribution of the immune activity in esophageal cancer.

**Figure 7:**
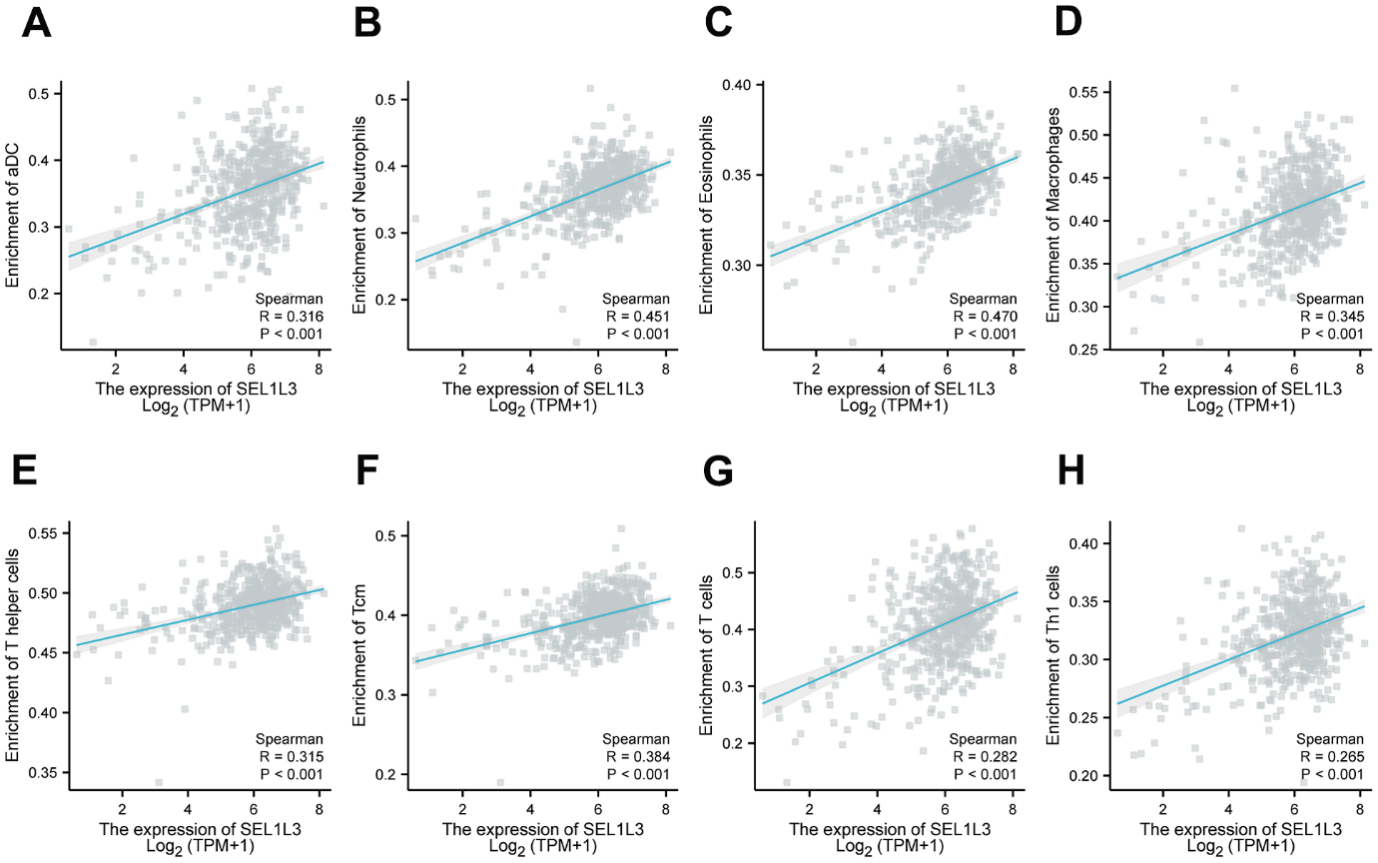
**The relationship between the infiltration of immune cells and SEL1L3 was examined through the utilization of scatter plots.** (**A**) aDC cells, (**B**) neutrophils cells, (**C**) eosinophils cells, (**D**) macrophages cells, (**E**) T helper cells, (**F**) Tcm cells, (**G**) T cells, (**H**) Th1 cells.

Figure 6A illustrates the presence of immune cells in patients with Esca, categorized based on low and high expression levels of SEL1L3. The correlation between the immune cells was calculated simultaneously (Figure 6B). Differential immune cell infiltration analysis revealed that high SEL1L3 expression was significantly positively correlated with the infiltration of antibody-drug conjugates (aDC), B cells, eosinophils, interstitial dendritic cells (iDC), macrophages, neutrophils, T cells, T helper (Th) cells, central memory CD8 T cells (Tcm), T follicular helper (TFH), Th1 cells, Th17 cells, and regulatory T cells (Tregs), and negatively correlated with CD8 T cells, natural killer (NK) cells, plasmacytoid dendritic cells (pDC), and M0 macrophages (Figure 6C). A heatmap of the infiltration of immune cells in the low- and high-SEL1L3 expression groups (Figure 6D). The correlation between the expression of SEL1L3 and each individual immune cell type was calculated (Figure 7).

### Role of SEL1L3 on ErbB/PI3K/mTOR signaling pathway

**Figure 8:**
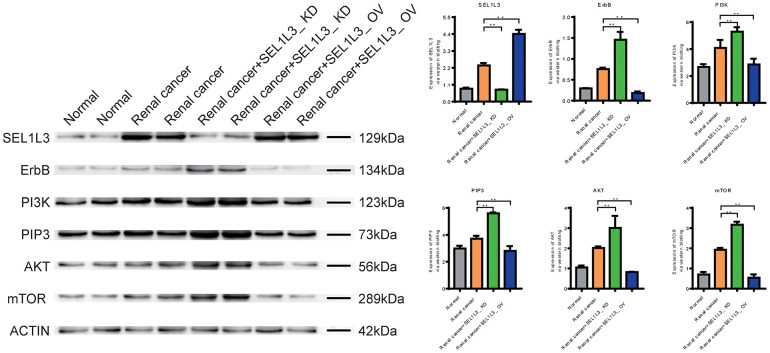
Experimental investigation into the impact of SEL1L3 on the ErbB/PI3K/mTOR signaling pathway.

Western blotting revealed that SEL1L3 expression was upregulated in the RC group. The core proteins (including ErbB, PI3K, PIP3, AKT, and mTOR) involved in ErbB signaling were also upregulated in the RC group. By knocking down SEL1L3, core proteins of the ErbB/PI3K/mTOR signaling pathway were upregulated in RCC cells (Figure 8).

### Role of SEL1L3 on apoptosis, invasion, cell cycle, and inflammation

**Figure 9:**
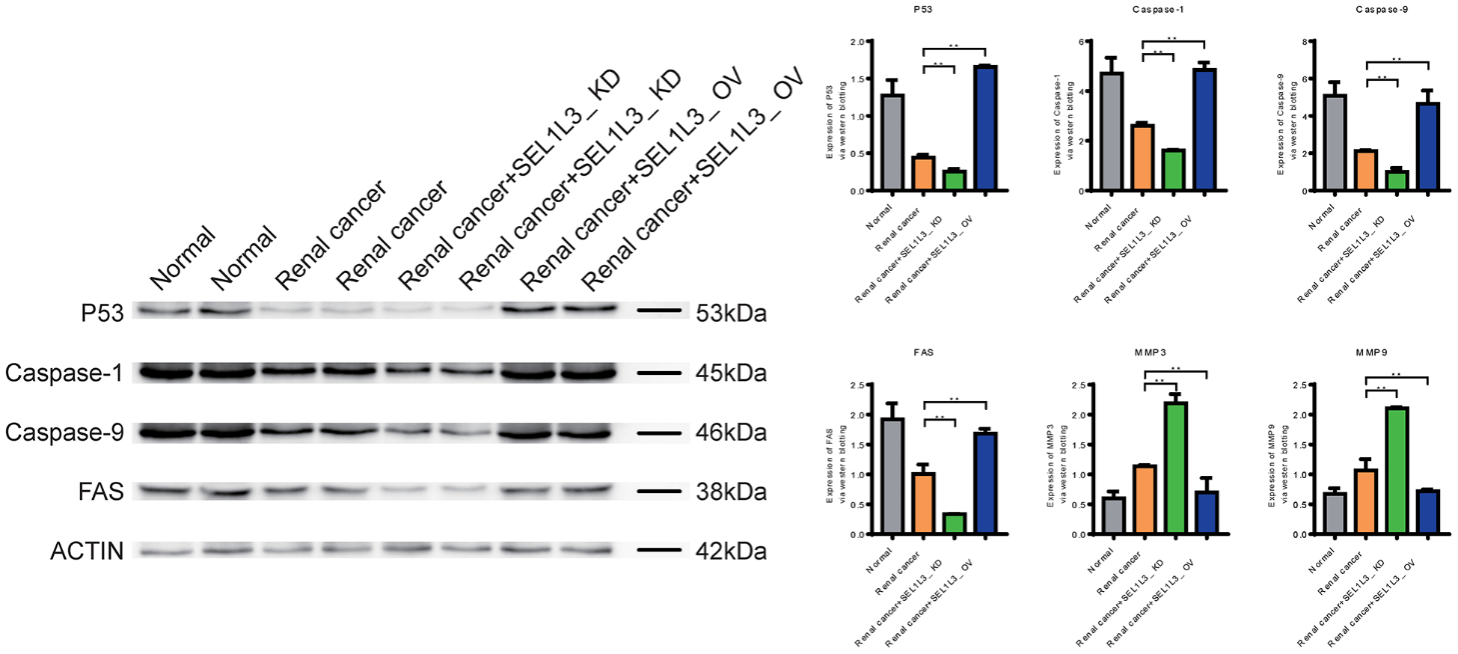
Experimental investigation into the impact of SEL1L3 on cell apoptosis function.

**Figure 10:**
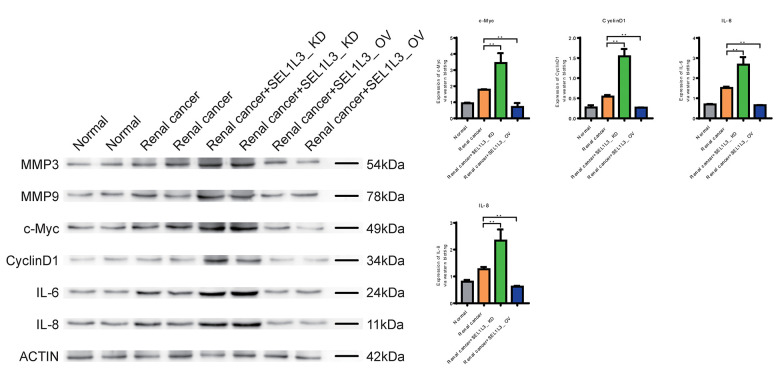
Experimental investigation into the impact of SEL1L3 on invasion, cell cycle and inflammation function.

Downregulation of Caspase-1, Caspase-9, and FAS expression in the RC group suggested that apoptosis was inhibited (Figure 9). Knockdown of SEL1L3 also resulted in the downregulation of apoptosis-related genes, whereas overexpression of SEL1L3 led to their upregulation. Biomarkers of invasion (MMP3 and MMP9), cell cycle (c-Myc and CyclinD1) and inflammation (IL-6 and IL-8) were significantly upregulated in the RC group (Figure 10). SEL1L3 knockdown enhanced this effect.

## DISCUSSION

Among the characteristics of kidney cancer with no clinical signs, kidney cancer in patients may be overlooked [18, 19]. The highly vascularized architecture of RCC is believed to promote hematogenous dissemination and the formation of distant metastases [20, 21]. In cases of early stage RCC, partial nephrectomy is typically employed as the conventional method for the excision of localized RCC with a favorable prognosis [22]. However, it is estimated that approximately 25–30% of patients diagnosed with kidney cancer exhibit metastases because most cases of kidney cancer manifest initially with a clinically silent course [23]. Angiogenesis of atherosclerotic plaques is intricately linked to the advancement and vulnerability of plaques [24, 25]. Consequently, pro-atherogenic alterations may precede and predispose patients to the onset of atherosclerosis. This study found that SEL1L3 is highly expressed in atherosclerotic plaques and kidney cancer, which may play a role in linking these two diseases.

SEL1L3, a member of the SEL1L (Sel-1 Suppressor of Lin-12-Like) family, is situated within the endoplasmic reticulum (ER) and plays a pivotal role in enabling ER-associated degradation. This degradation process is triggered by ER stress, which promotes the breakdown of misfolded proteins [26].

SEL1L3 was aberrantly expressed in human brain microvascular endothelial cells after exposure to HIV-1 CRF02_AG, suggesting its potential involvement of SEL1L3 in the pathophysiological mechanisms of microvessel function. SEL1L3 has been implicated in the development of several types of cancer. Previous studies have documented SEL1L3 is a promising prognostic indicator in colorectal cancer, melanoma, and lung adenocarcinoma [26–28]. In this study, SEL1L3 was found to be overexpressed in renal cancer cells and atherosclerotic plaque tissue, and may be involved in the progression of RCC.

In the current study, SEL1L3 overexpression was linked to aggressive behavior but led to better survival in patients with RCC. However, this seems to be contradictory. Cao et al. [29] observed similar results; they found that CXCL11 was highly differentially expressed in colon adenocarcinoma (COAD), but played a significant defensive role in the development of COAD and contributed to a more favorable prognosis for patients diagnosed with COAD. Cao et al. proposed that this result may be because CXCL11 can cause the recruitment of DC, NK cells, and T cells, which play important roles in inhibiting tumor growth and improving prognosis. In this study, SEL1L3 knockdown in renal RCC cells activated the ErbB/PI3K/mTOR signaling pathway, inhibited apoptosis, and promoted inflammation. This may result in better survival of patients with low SEL1L3 expression.

Moreover, experimental evidence has revealed that the inhibition of SEL1L3 expression leads to a reduction in the proliferation of renal cancer cells and triggers apoptosis, suggesting that SEL1L3 could potentially serve as a viable therapeutic target for renal cancer. SEL1L3 expression can effectively predict RC prognosis The development of drugs targeting SEL1L3 may benefit patients with RC and atherosclerosis.

This study has some limitations. These clinical results are insufficient to support our research results. However, the underlying mechanism of SEL1L3 action has not yet been explored. Further research should investigate the mechanism of action of SEL1L3 in RC and atherosclerosis.

In summary, the association between SEL1L3 and renal cancer highlights the potential significance of SEL1L3 in the pathogenesis of this disease and suggests that targeting SEL1L3 may be a promising approach for the treatment of renal cancer.

## CONCLUSIONS

SEL1L3 is overexpressed in RCC and atherosclerotic plaques, and is correlated with several biological processes in RCC. The utilization of SEL1L3 as a molecular target holds potential for the timely detection and accurate therapeutic intervention of RCC, while also offering valuable insights into the underlying association between cancer and atherosclerosis.

## MATERIALS AND METHODS

### Data retrieval

We searched for an atherosclerotic plaque expression matrix in the Gene Expression Omnibus (GEO) database (http://www.ncbi.nlm.nih.gov/geo/). The atherosclerosis dataset GSE28829 based on the GPL570 platform contained 13 normal tissues and 16 atherosclerotic plaque tissues, and GSE43292 stockpiled on the GPL6244 platform consisted of 32 normal tissues and 32 atherosclerotic plaque tissues. The RNA sequencing data of RCC patients were downloaded from The Cancer Genome Atlas (TCGA) data portal (https://tcga-data.nci.nih.gov/tcga/), and included 72 normal and 535 tumorous tissues.

### Differential expression analysis

Gene differential analysis was conducted in the three datasets using the “Limma” package in R 3.6.1 software. Statistical significance was set at P < 0.05 and absolute log fold change (FC) > 1. Venn plots were constructed to overlap common differential expressed genes in the three datasets.

### Volcano plot of gene expression

Volcano plots were constructed using the “ggplot2” package in R software to visualize gene expression in the three datasets.

### Protein-protein interaction (PPI) network

The Search Tool for the Retrieval of Interacting Genes (STRING) database (https://www.string-db.org) was used to construct a PPI network of the differentially expressed genes (DEGs). Visualization of this network was performed using Cytoscape software (https://cytoscape.org/).

### Functional enrichment analysis

Gene Ontology (GO) and Kyoto Encyclopedia of Genes and Genomes (KEGG) pathway enrichment analyses were performed using the DAVID (https://david.ncifcrf.gov/) and Metascape (https://metascape.org) platforms. Functional enrichment analysis is a computational approach used to determine whether a specific collection of genes or proteins is enriched for specific biological functions, pathways, or processes. This analytical method has been widely employed in genomics and proteomics to obtain a deeper understanding of the molecular mechanisms underlying experimental findings.

### Gene set enrichment analysis (GSEA)

GSEA was performed to evaluate enriched KEGG pathways using GSEA software (http://www.broadinstitute.org/gsea). We normalized the gene expression data and then filtered out the low-expressed genes to reduce noise.

### Survival analysis

Based on the TCGA and Genotype-Tissue Expression (GTEx) data, the Gene Expression Profiling Interactive Analysis (GEPIA) database (http://gepia.cancer-pku.cn/) was used for survival analysis.

### Heat map of gene expression

The R package heatmap facilitated the creation of a visually informative heatmap effectively depicting variations in the expression levels of core genes among RCC and normal tissue samples in the GSE14762 and GSE53757 datasets.

### Immune infiltration analysis

The estimation of immune cell infiltration in RCC was conducted using the “CIBERSORT” R package, which uses a gene set containing specific markers of immune cells to calculate the enrichment score. Samples with a significance level of confidence and a cutoff of p < 0.05 were selected.

### Verification of the role of SEL1L3

The HEK-293 and Caki-1 cell lines were obtained from the National Biomedical Experimental Cell Resource Bank in Beijing, China. Cells were divided into four groups: control (normal renal cancer cells), RCC (renal cancer cells), RCC/SEL1L3-KD (RCC with SEL1L3 knockdown), and RCC/SEL1L3-OV (RCC with SEL1L3 overexpression). Sample preparation involves the extraction and purification of the protein of interest from a sample, which may include processes such as homogenization, cell lysis, and chromatography, depending on the nature of the sample. For protein separation, the extracted proteins were separated based on molecular weight using sodium dodecyl sulfate-polyacrylamide gel electrophoresis (SDS-PAGE). For protein transfer, following separation, the proteins were transferred from the gel medium onto polyvinylidene fluoride (PVDF) membranes through electroblotting. This step entails the immobilization of proteins onto the membrane. To prevent non-specific binding of the primary antibody, the membrane is subjected to incubation with a blocking solution, typically consisting of 5% non-fat dry milk or 1% bovine serum albumin (BSA) in Tris-buffered saline (TBS) with 0.1% Tween-20 (TBST) for approximately 1 h at room temperature or overnight at 4° C. Subsequently, the membrane is incubated with a primary antibody that specifically targets the protein of interest, diluted in the aforementioned blocking solution, for approximately 1 h at room temperature or overnight at 4° C. After washing, the membrane was incubated with secondary antibodies, wherein an enzyme such as horseradish peroxidase (HRP) or alkaline phosphatase (AP) was conjugated to the secondary antibody. This conjugated enzyme recognizes and binds primary antibodies. Subsequently, the membrane was washed again to eliminate any unbound secondary antibody. For signal detection, the membrane was exposed to a substrate solution specific to an enzyme conjugated to a secondary antibody. This process yields a chemiluminescent or chromogenic signal that can be detected using an X-ray film or specialized imaging system. The signal intensity was directly proportional to the quantity of protein in the sample. Quantification of the signal intensity can be achieved by employing image analysis software, which enables the comparison of results across various samples or treatments.

### Data availability statement

The datasets generated during and/or analyzed during the current study are available from the corresponding author on reasonable request.
